# Prognostic Significance of Tumor and Inflammatory Markers in Disease-Free and Overall Survival Duration in Colonic Adenocarcinoma Patients

**DOI:** 10.7759/cureus.68667

**Published:** 2024-09-04

**Authors:** Mehmet Berksun Tutan, Kaan Canal, Orhan Aslan, İsmail Sezikli, Mahmut Arif Yüksek, Ramazan Topçu, Veysel Barış Turhan, Murat Kendirci, İbrahim Tayfun Şahiner

**Affiliations:** 1 Department of General Surgery, Alaca State Hospital, Çorum, TUR; 2 Department of General Surgery, Hitit University Faculty of Medicine, Çorum, TUR; 3 Department of General Surgery, Medical Point Hospital, Gaziantep, TUR

**Keywords:** carbohydrate antigen 125 (ca125), carcinoembryonic antigen (cea), overall survival (os), disease-free survival (dfs), tumor markers, prognosis, colonic adenocarcinoma

## Abstract

Introduction

Colorectal carcinoma (CRC) continues to be a major global health concern, contributing substantially to cancer incidence and mortality. Colonic adenocarcinoma, a common subtype of CRC, is influenced by various prognostic factors, including tumor stage, histopathological characteristics, and tumor markers. Despite their routine use in clinical settings, the prognostic value of traditional tumor markers, such as carcinoembryonic antigen (CEA), carbohydrate antigen 19-9 (CA 19-9), and others, is still under debate. In this study, we aim to analyze the tumor markers’ prognostic significance in our CRC patients in terms of disease-free survival and overall survival.

Methods

A retrospective study was conducted on 71 patients who underwent surgery for colonic adenocarcinoma between January 1, 2018, and January 1, 2024. Data on patient demographics, recurrence rates, survival times, and tumor marker levels (CEA, CA 19-9, CA 125, AFP, and CRP to albumin ratio (CAR)), disease-free survival duration (DFS), and overall survival durations (OS) were collected and analyzed. Statistical analyses included Pearson and Spearman correlation coefficients, the Mann-Whitney U test, ROC curve analysis, and Kaplan-Meier survival analysis.

Results

The study found that elevated CAR and CA 125 levels were significantly associated with higher mortality and recurrence rates, whereas elevated CEA levels were strongly predictive of recurrence. Receiver operating characteristic (ROC) analysis identified optimal cutoff values for these markers, with CEA ≥ 47.145, CA 125 ≥ 15.85, and CAR ≥ 6.796 demonstrating high specificity and predictive value for recurrence. Kaplan-Meier analysis revealed that patients with CEA < 47.145 had a significantly longer DFS (67.7 months) compared to those with CEA ≥ 47.145 (24 months, p < 0.001). Similarly, patients with CA 125 < 15.85 and CAR < 6.796 showed longer DFS compared to those with higher values. Overall survival analysis also highlighted that patients with CA 125 < 21.71 and CAR < 4.09 had better survival outcomes, with significant differences of 26 and 10 months, respectively (p < 0.001 and p = 0.001).

Conclusion

Tumor markers, such as CEA, CA 125, and CAR, hold significant prognostic value in colonic adenocarcinoma, with higher levels correlating with poorer outcomes. These findings underscore the importance of integrating tumor markers into clinical decision-making to optimize treatment strategies and improve patient survival. Future research should focus on standardizing the use of these markers and exploring novel biomarkers for enhanced prognostication.

## Introduction

Colorectal carcinoma (CRC) is one of the most prevalent and lethal malignancies worldwide, exerting a significant impact on public health [1]. Accounting for around 10% of all cancer diagnoses and deaths, CRC poses a major challenge to healthcare systems, highlighting the need for ongoing research and advancements in its management [2]. The prognosis of colonic adenocarcinoma, a common subtype of colorectal cancer, is influenced by a complex interplay of factors, including tumor stage, histopathological features, genetic mutations, and patient-specific variables, such as age and comorbidities [3]. Among these, tumor markers have emerged as a focal point of interest for their potential prognostic utility [3].

Tumor markers, which encompass a variety of proteins and molecules secreted by cancer cells or produced by the body in response to cancer, are employed in clinical practice to aid in the early detection, monitoring, and prognostication of colonic adenocarcinomas [4-6]. Commonly utilized tumor markers in this context include carcinoembryonic antigen (CEA), carbohydrate antigen 19-9 (CA 19-9), and, to a lesser extent, markers such as tissue polypeptide antigen (TPA) and circulating tumor DNA (ctDNA) [4-7]. Elevated levels of these markers have been associated with increased tumor burden, metastatic potential, and overall disease aggressiveness [7,8].

The prognostic significance of tumor markers in colonic adenocarcinoma, particularly their capacity to predict disease-free survival (DFS) and overall survival (OS), represents a critical area of investigation [3,4,8]. DFS, defined as the duration following primary treatment during which a patient remains free from any signs or symptoms of cancer, is a key endpoint in evaluating the efficacy of therapeutic interventions [9]. OS, which measures the time from diagnosis or the initiation of treatment until death from any cause, remains the gold standard for assessing long-term outcomes in cancer patients [9]. Identifying reliable biomarkers that accurately predict these outcomes is crucial for personalizing treatment regimens and improving patient prognoses [3].

To this end, the C-reactive protein-to-albumin ratio (CAR) has emerged as a valuable prognostic marker in various malignancies, such as colonic adenocarcinoma [5]. By reflecting both systemic inflammation and nutritional status, CAR offers an insight into a patient's overall state [5]. Elevated CAR levels have been associated with poorer disease-free and OS durations, making it an important parameter in the prognostic evaluation of colonic adenocarcinoma patients [5]. Integrating CAR into clinical practice can enhance the accuracy of predictions.

Despite the widespread use of tumor markers such as CEA and CA 19-9 in clinical practice, their prognostic value remains a subject of ongoing debate [10,11]. Numerous studies have examined the relationship between preoperative and postoperative levels of these markers and patient outcomes, yielding inconsistent results [10-12]. For example, elevated preoperative CEA levels have been correlated with poorer survival rates and higher recurrence risks, suggesting a potential role in risk stratification [13,14]. However, the heterogeneity of study designs, patient populations, and methodologies has led to inconsistent findings, underscoring the need for standardized research approaches [10,11].

Advancements in molecular biology and technology have introduced novel tumor markers and assays that promise enhanced sensitivity and specificity [4,7]. For instance, ctDNA has emerged as a promising biomarker for detecting minimal residual disease and early recurrence, offering potential advantages over traditional markers [4,7]. The integration of these novel biomarkers into clinical practice could enhance our understanding of colonic carcinoma management, enabling more precise prognostication and timely interventions [7]. However, the high cost and limited accessibility of these newer biomarkers, particularly in rural areas, underscore the continued importance of widely available tumor markers. In this study, we aim to evaluate the prognostic significance of tumor markers in our cohort of colorectal carcinoma patients, with a focus on DFS and OS.

## Materials and methods

The study was conducted following approval from the Hitit University Clinical Research Ethics Committee (date: 03.04.2024, Decision Number: 2024-08), with all procedures involving human participants adhering strictly to the ethical standards set by the institution and the 1964 Declaration of Helsinki.

Between January 2018 and January 2024, 71 patients who underwent surgery for colonic adenocarcinoma in Hitit University Faculty of Medicine, Department of General Surgery, and had no medication use, hematologic diseases, or active infections that would alter hemogram and biochemistry parameters were retrospectively included in the study. Those with prior oncological diseases other than colonic carcinoma were excluded. The data collected included age, gender, comorbidities, recurrence and mortality rates, disease-free survival, overall survival, observation periods, hemogram and biochemistry results, and preoperative values for C-reactive peptide (CRP) to albumin ratio (CAR), mean platelet volume (MPV), and tumor markers, all of which were obtained from the hospital’s database.

This study was retrospectively designed. Statistical analyses were carried out using IBM SPSS Statistics for Windows software (version 26; IBM Corp., Armonk, NY). Descriptive statistics were used to report categorical variables as frequencies and percentages, while continuous variables were presented as mean ± standard deviation for normally distributed data or as median (range) for non-normally distributed data. The Shapiro-Wilks test was applied to assess the normality of data distribution. Correlations between variables were analyzed using Pearson or Spearman correlation coefficients, depending on the distribution of the data. The Mann-Whitney U test was used to compare non-normally distributed numerical variables, such as survey duration, disease survival duration, disease-free survival duration, overall survival duration, neutrophil count, CA 19-9, CEA, CA 125, AFP, and CAR, between independent groups. Student’s t-test was utilized to compare normally distributed variables, including age, MPV, and albumin levels, across groups. Categorical variables such as gender, presence of additional systemic diseases, adjuvant therapy, recurrence, and mortality rates were analyzed using the chi-square test.

Receiver operating characteristic (ROC) curves were generated to evaluate the discriminative power of tumor markers. Cutoff values for these markers were established using the area under the curve (AUC) and the Youden index. Sensitivity, specificity, positive predictive value (PPV), negative predictive value (NPV), and accuracy were calculated based on these cutoff values, and odds ratios were computed accordingly. Kaplan-Meier survival analyses were conducted to assess disease-free and overall survival in relation to tumor marker cutoffs, with statistical significance between groups determined by the log-rank test. The Haldane-Anscombe correction was applied to address instances where odds ratios approached infinity. A p-value of less than 0.05 was considered statistically significant.

## Results

A total of 71 patients were enrolled in the study, with follow-up durations ranging from 18 to 72 months. Among these patients, 45 (63.4%) were male, and 26 (36.6%) were female. Additionally, 43 patients (60.56%) had concomitant systemic diseases, including diabetes mellitus (DM), hypertension (HT), and asthma. During the study period, 10 patients died, leaving 61 patients (85.9%) alive at the time of the final observation.

Comparison of variables between alive and deceased patient groups

There was no significant difference in the distribution of gender between the deceased and surviving patients (p = 0.811). The mean age of deceased patients was 74.5 ± 12.27 years, significantly higher than that of the surviving patients (p = 0.026). No significant differences were observed between the two groups regarding additional systemic diseases, cancer stage, or the requirement for adjuvant therapy (p = 0.296, p = 0.439, and p = 0.502, respectively). The recurrence rate was notably higher among deceased patients (60.0%) compared to survivors (8.2%, p < 0.001). There were no significant differences in survey duration between the two groups (p = 0.091). The median disease-free survival duration of patients who were still alive was longer at 48 months than that of deceased patients (p < 0.001). No significant differences were noted between the groups for neutrophil count, MPV, or albumin levels (p = 0.130, p = 0.461, and p = 0.699). However, the median CRP level was significantly higher in the deceased group compared to the surviving group (p = 0.003). When tumor markers were evaluated, CA 19-9, CEA, and AFP levels were not significantly different between surviving and deceased patients; however, CA 125 and CAR levels were significantly higher in deceased patients (p = 0.568, p = 0.122, p = 0.122, p = 0.698, p = 0.004, and p = 0.001, respectively) (see Table 1).

**Table 1:** Univariate comparisons between groups according to mortality MPV: mean platelet volume, CRP: C-reactive peptide, CA 19-9: carbohydrate antigen 19-9, CEA: carcinoembryonic antigen, CA 125: cancer antigen 125, AFP: alpha-fetoprotein, CAR: CRP albumin ratio

Variables	Subgroups	Alive (n=61)	Deceased (n=10)	Statistical Significance
Gender	Female	22 (36.07%)	4 (40%)	0.811
	Male	39 (63.93%)	6 (60%)	
Age		67±9.24	74.5±12.27	0.026
Existence of Additional Systemic Diseases		35 (57.38%)	8 (80%)	0.296
Stage	I	6 (9.84%)	1 (10%)	0.439
	II	31 (50.82%)	3 (30%)	
	III	24 (39.34%)	6 (60%)	
Adjuvant Therapy		34 (55.74%)	7 (70%)	0.502
Recurrence Rate		5 (8.2%)	6 (60%)	<0.001
Survey Duration		48 (18-72)	36 (18-60)	0.091
Diseased Survival Duration		0 (0-30)	9 (0-48)	<0.001
Disease-Free Survival Duration		48 (12-72)	18 (6-48)	<0.001
Overall Survival Duration		48 (18-72)	36 (18-60)	0.089
Neutrophil Count		4.59 (1.91-14.15)	5.53 (2.49-10.81)	0.130
MPV		9.79±1.23	9.49±1.01	0.461
CRP		15.79 (3.13-29.84)	24.05 (13.38-66.95)	0.003
Albumin		3.79±0.45	3.73±0.51	0.699
CA 19-9		14.35 (0.2-64)	13.72 (1-27)	0.568
CEA		2.59 (0.5-593)	4.77 (1.43-319)	0.122
CA 125		12.8 (1-243.7)	27.84 (5-109.7)	0.004
AFP		2.4 (0.3-10.6)	2.44 (1.3-5.98)	0.698
CAR		4.1 (0.7-9.6)	7.7 (4.1-15.2)	0.001

Comparison of variables between no-recurrence and recurrent disease patient groups

During the study, recurrence was observed in 11 patients, while 60 patients were documented as disease-free at the postoperative follow-up. Similar to mortality, no gender-related differences or differences in additional systemic diseases were found between the recurrence groups (p = 0.516). In contrast to mortality, age medians did not differ between recurrence groups (p = 0.502). The use of adjuvant chemotherapy varied between groups, which was attributed to the necessity in the presence of recurrence (p = 0.002; see Table 2).

**Table 2:** Univariate comparisons between groups according to recurrence MPV: mean platelet volume, CRP: C-reactive peptide, CA 19-9: carbohydrate antigen 19-9, CEA: carcinoembryonic antigen, CA 125: cancer antigen 125, AFP: alpha-fetoprotein, CAR: CRP albumin ratio

Variables	Subgroups	No Recurrence	Recurrent Disease	Statistical Significance
Gender	Female	21 (35%)	5 (45.45%)	0.516
	Male	39 (65%)	6 (54.55%)	
Age		68.4±9.98	66.18±10.21	0.502
Existence of Additional Systemic Diseases		37 (61.67%)	6 (54.55%)	0.657
Stage	I	7 (11.67%)	0 (0%)	0,071
	II	31 (51.67%)	3 (27.27%)	
	III	22 (36.67%)	8 (72.73%)	
Adjuvant Therapy		30 (50%)	11 (100%)	0.002
Recurrence Rate				
Mortality Rate		4 (6.67%)	6 (54.55%)	<0.001
Survey Duration		48 (18-72)	36 (24-60)	0.033
Diseased Survival Duration		0 (0-6)	12 (0-48)	<0.001
Disease-Free Survival Duration		48 (18-72)	18 (6-24)	<0.001
Overall Survival Duration		48 (18-72)	36 (24-60)	0.032
Neutrophil Count		4.47 (1.91-14.15)	5.83 (3.01-10.81)	0.028
MPV		9.77±1.25	9.67±0.92	0.816
CRP		16.04 (3.13-44.26)	17.7 (10.15-66.95)	0.068
Albumin		3.78±0.45	3.79±0.51	0.999
CA 19-9		14.13 (0.2-64)	15 (0.2-27)	0.600
CEA		2.47 (0.5-74)	51.87 (2.14-593)	0.001
CA 125		12.75 (1-243.7)	23 (5-79.6)	0.010
AFP		2.4 (0.3-10.6)	2.27 (0.5-3.1)	0.567
CAR		4.3 (0.7-11.6)	4.9 (2.6-15.2)	0.045

The mortality rate during the observation period was 6.67% in the no-recurrence group and 54.55% in the recurrent disease group, showing a statistically significant difference (p < 0.001). Overall survival duration differed significantly between the groups, with a median of 48 months (range: 18-72) in the no-recurrence group versus 36 months (range:: 24-60) in the recurrence group (p = 0.033). Disease-free survival duration was also significantly longer in the no-recurrence group, with a median of 48 months (range: 18-72) compared to 18 months (range: 6-24) in the recurrent disease group (p < 0.001).

Neutrophil count was significantly higher in the recurrent disease group, with a median of 5.83 (range: 3.01-10.81) compared to 4.47 (range: 1.91-14.15) in the no-recurrence group (p = 0.028). MPV, CRP, and albumin levels did not show significant differences between the groups (p = 0.816, p = 0.068, and p = 0.999, respectively).

Assessment of tumor markers revealed that CA 19-9 levels were 14.13 (range: 0.2-64) in the no-recurrence group and 15 (range: 0.2-27) in the recurrence group, with no significant difference between groups (p = 0.600). CEA levels were significantly higher in the recurrence group, with a median of 51.87 (range: 2.14-593) compared to 2.47 (range: 0.5-74) in the no-recurrence group (p = 0.001). CA 125 levels were also elevated in the recurrence group, with a median of 23 (range: 5-79.6) versus 12.75 (range: 1-243.7) in the no-recurrence group (p = 0.010). AFP levels did not differ significantly between groups (2.4, range: 0.3-10.6 in the no-recurrence group, vs. 2.27, range: 0.5-3.1 in the recurrence group, p = 0.567). CAR was significantly higher in the recurrence group (median: 4.9, range: 2.6-15.2) compared to the no-recurrence group (median: 4.3, range: 0.7-11.6) (p = 0.045; see Table 1).

Evaluation of optimal cutoff points for prediction of recurrence

**Figure 1:**
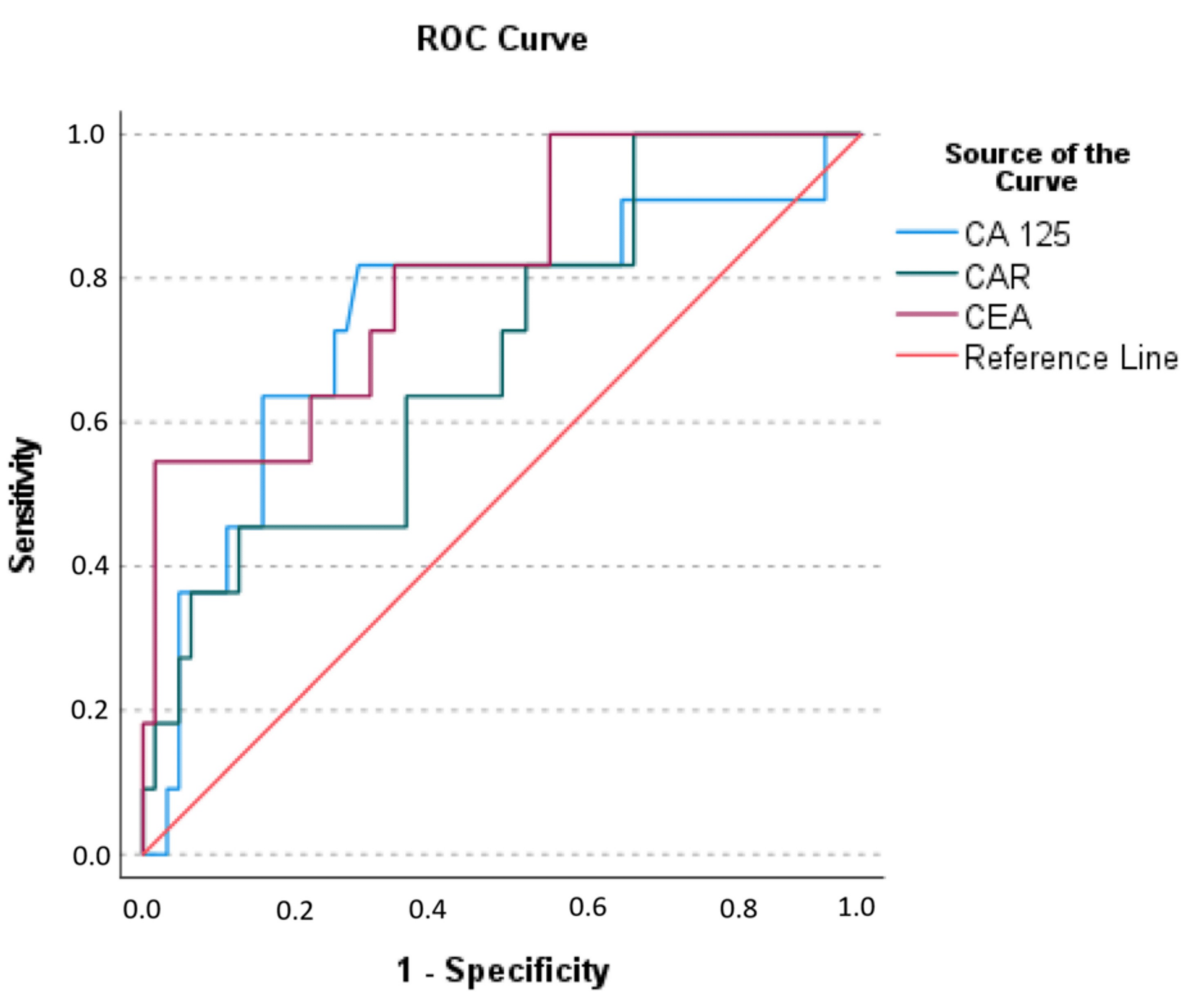
Receiver operating curve of CEA, CA 125, and CAR for recurrence CA 125: cancer antigen 125, CEA: carcinoembryonic antigen, CAR: CRP-to-albumin ratio, ROC: receiver operating curve

ROC analysis was employed to determine the optimal values of CEA, CA 125, and CAR for distinguishing between recurrence and no-recurrence groups (Figure 1).

For the best distinction of recurrence, the optimal CEA cutoff value was determined to be ≥ 47.145 with 54.5% sensitivity, 98.3% specificity, 85.7% positive predictive value, 92.2% negative predictive value, and 91.5% test accuracy (OR: 70.8, 95% CI: 7.060-710.032, p < 0.001). The optimal CA 125 cutoff value was found to be ≥ 15.85 with 81.8% sensitivity, 70% specificity, 33.3% positive predictive value, 95.5% negative predictive value, and 71.8% test accuracy (OR: 10.5, 95% CI 2.060-53.517, p = 0.001). Similarly, the optimal CAR cutoff value was ≥ 6.796 with 45.5% sensitivity, 86.7% specificity, 38.5% positive predictive value, 89.7% negative predictive value, and 89.7% test accuracy (OR: 5.417, 95% CI: 1.334-21.986, p = 0.011; see Table 3).

**Table 3:** Diagnostic parameters of tumor marker cutoff values for the distinction of recurrence PPV: positive predictive value, NPV: negative predictive value, AUC: area under the curve, SE: standard error, CI: confidence interval, p: statistical significance, OR: odds ratio, CEA: carcinoembryonic antigen, CA125: cancer antigen 125, CAR: C-reactive protein/albumin ratio

Variables	Cut-off (for recurrence)	Diagnostic Values					ROC Analysis			Odds Ratio		
		Sensitivity	Specificity	PPV	NPV	Accuracy	AUC (SE)	95% CI	p	OR	95% CI	p
CEA	≥47.145	54.5%	98.3%	85.7%	92.2%	91.5%	0.809 (0.071)	0.670-0.949	<0.001	70.800	7.060-710.032	<0.001
CA 125	≥15.85	81.8%	70.0%	33.3%	95.5%	71.8%	0.745 (0.090)	0.568-0.921	0.007	10.500	2.060-53.517	0.001
CAR	≥6.796	45.5%	86.7%	38.5%	89.7%	80.3%	0.691 (0.085)	0.525-0.857	0.024	5.417	1.334-21.986	0.011

Evaluation of the optimal cutoff points for the prediction of overall mortality

**Figure 2:**
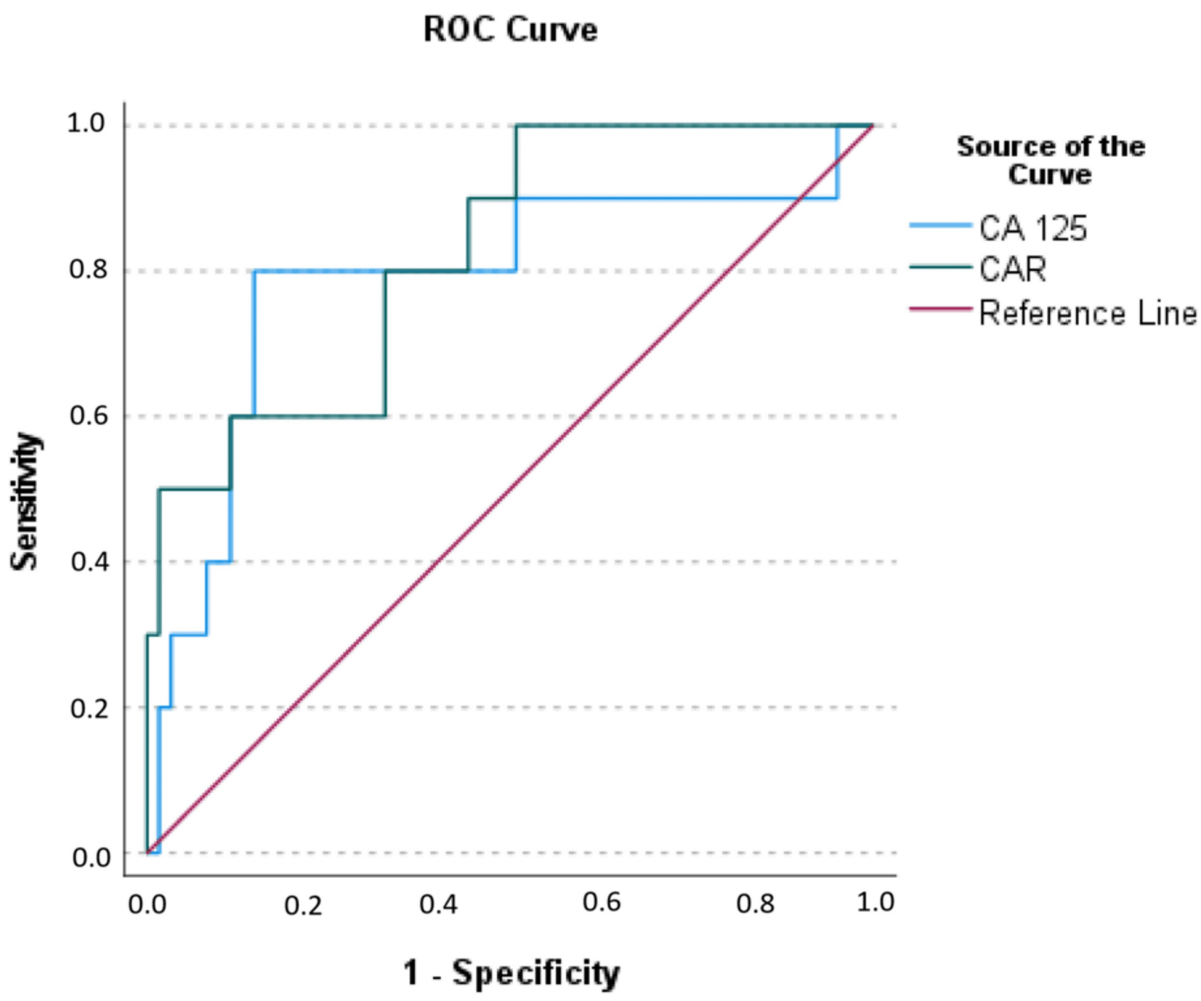
Receiver operating curve of CA 125 and CAR for mortality CA 125: cancer antigen 125, CAR: C-reactive protein/albumin ratio, ROC: receiver operating curve

To assess the optimal values of CA 125, and CAR for distinguishing between no-recurrence and recurrence groups, the area under the curve and the Youden index were employed in ROC analysis again (Figure 2).

For the best distinction of mortality, the optimal CA 125 cutoff value was determined to be ≥ 21.715, with 80% sensitivity, 85.2% specificity, 47.1% positive predictive value, 96.3% negative predictive value, and 84.5% test accuracy (OR: 23.111, 95% CI: 4.208-126.941, p < 0.001). The optimal CAR cutoff value was ≥ 4.09 with 95.5% sensitivity, 49.2% specificity, 24.4% positive predictive value, 98.4% negative predictive value, and 56.3% test accuracy (OR: 20.333, 95% CI: 8.177-50.558, p = 0.004) (Table 4).

**Table 4:** Diagnostic parameters of tumor marker cutoff values for the distinction of mortality PPV: positive predictive value, NPV: negative predictive value, AUC: area under the curve, SE: standard error, CI: confidence interval, p: statistical significance, OR: odds ratio, CA125: cancer antigen 125, CAR: C-reactive protein/albumin ratio, *: Estimated with the Haldane-Anscombe correction technique

Variables	Cutoff (for Mortality)	Diagnostic Values					ROC Analysis			Odds Ratio		
		Sensitivity	Specificity	PPV	NPV	Accuracy	AUC (SE)	95% CI	p	OR	95% CI	p
CA 125	≥21.715	80.0%	85.2%	47.1%	96.3%	84.5%	0.787 (0.093)	0.605-0.969	0.002	23.111	4.208-126.941	<0.001
CAR	≥4.09	95.5%*	49.2%	24.4%	98.4%*	56.3%	0.825 (0.067)	0.693-0.957	<0.001	20.333*	8.177-50.558*	0.004

Survival analysis according to cutoff values for disease-free survival

**Figure 3:**
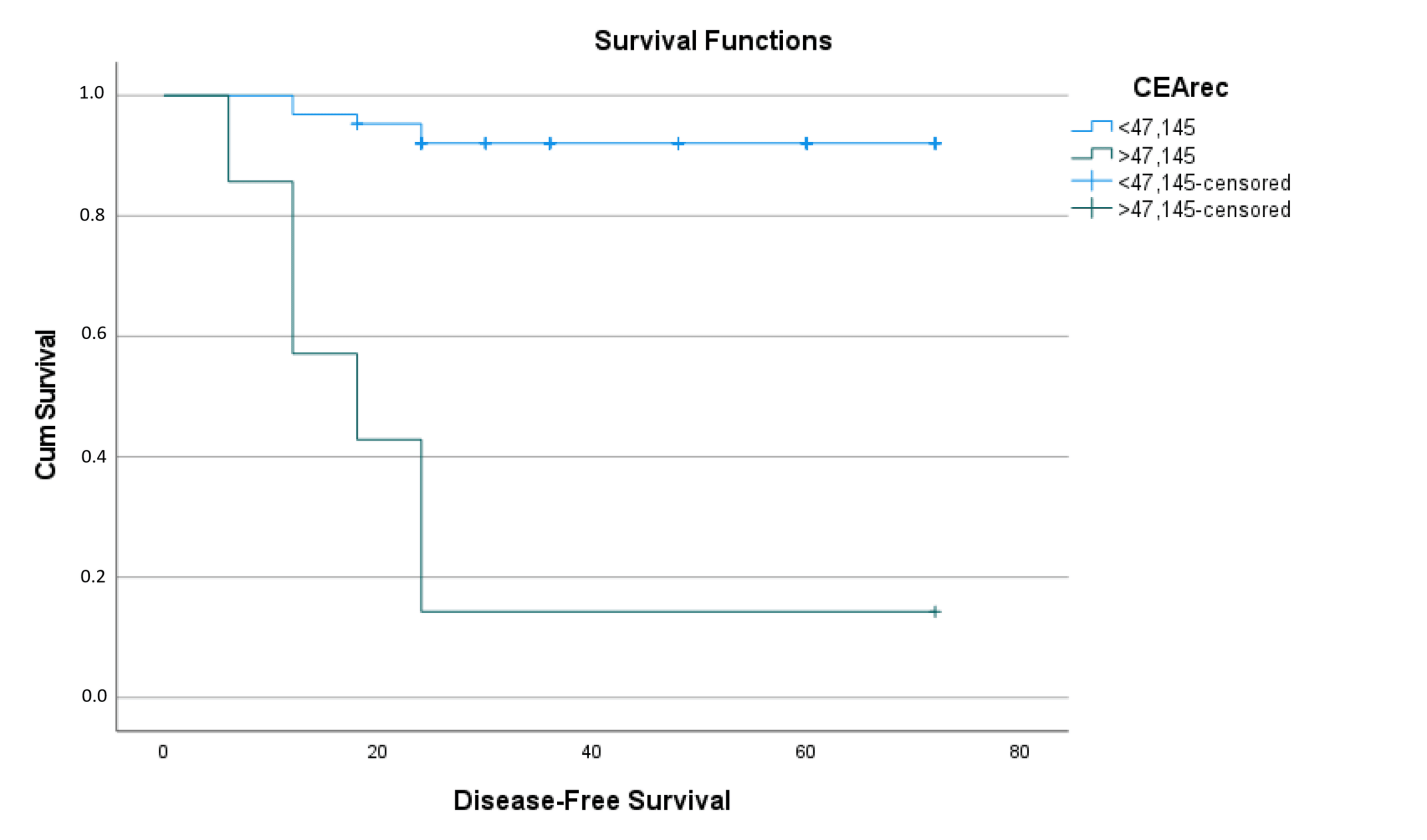
Kaplan-Meier survival analysis function graph of CEA for disease-free survival CEArec: Carcinoembryonic antigen cutoff groups for recurrence

The Kaplan-Meier survival analysis and the log-rank test indicated a mean estimated disease-free survival of 63.38 ± 2.40 months (95% CI: 58.668-68.090). Patients with CEA levels < 47.145 had a mean estimated disease-free survival time of 67.73 ± 1.84 months (95% CI: 64.124-71.34), while those with CEA ≥ 47.145 had a mean estimated disease-free survival time of 24 ± 7.76 months (95% CI: 66.864-72.77), showing a significant difference of approximately 43 months (p < 0.001) (Table 5). At 60 months, the disease-free survival rate was approximately 90% in the CEA-negative group, decreasing to less than 20% in the CEA-positive group (Figure 3).

**Table 5:** Estimated durations for disease-free survival stratified for CA 125, CAR, and CEA cutoff values CEA: carcinoembryonic antigen, CA 125: cancer antigen 125, CAR: C-reactive protein/albumin ratio, CI: confidence interval

Variables	Cutoff	Estimated Disease-Free Survival Duration (months)	Std. Error	95% CI	Log Rank test Statistical Significance
CEA for Disease-Free Survival	<47.145	67.73	1.84	64.124-71.34	<0.001
	≥47.145	24.00	7.76	8.787-39.21	
CA 125 for Disease-Free Survival	<15.85	69.82	1.51	66.864-72.77	<0.001
	≥15.85	52.72	5.26	42.406-63.03	
CAR for Disease-Free Survival	<6.796	66.35	2.19	62.057-70.64	0.008
	≥6.796	50.31	7.71	35.196-65.42	
Estimated Disease-Free Survival Duration (All Participants)	x	63.38	2.40	58.668-68.090	x

**Figure 4:**
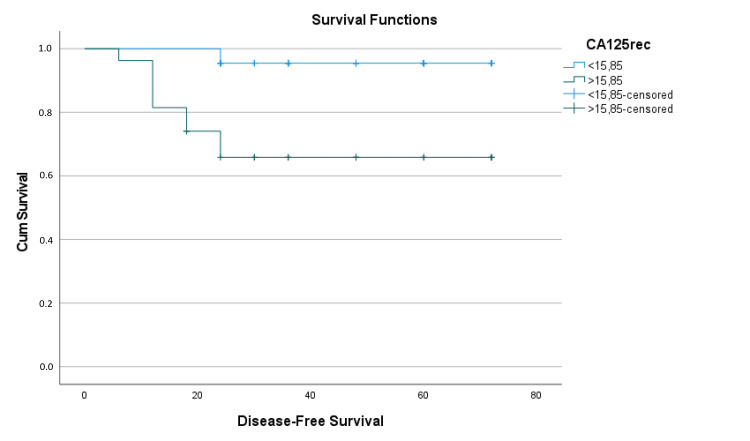
Kaplan-Meier survival analysis function graph of CA 125 for disease-free survival CA125rec: Cancer antigen 125 cutoff groups for recurrence

Patients with CA 125 levels < 15.85 had a mean estimated disease-free survival time of 69.82 ± 1.51 months (95% CI: 66.86-72.77), while those with CA 125 ≥ 15.85 had a mean estimated disease-free survival time of 52.72 ± 5.26 months (95% CI: 42.406-63.03), with a difference of approximately 17 months (p < 0.001) (Table 5 and Figure 4).

**Figure 5:**
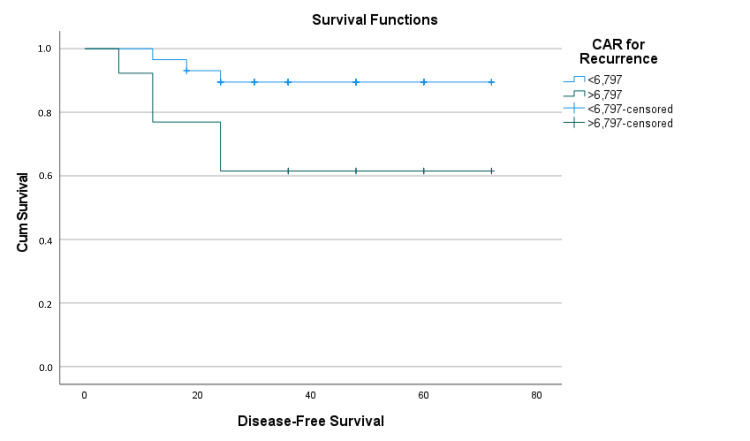
Kaplan-Meier survival analysis function graph of CAR for disease-free survival C-reactive protein/albumin ratio (CAR) cutoff groups for recurrence

Patients with CAR < 6.796 had a mean estimated disease-free survival time of 66.35 ± 2.19 months (95% CI: 62.057-70.64), while those with CAR ≥ 6.796 had a mean estimated disease-free survival time of 50.31 ± 7.71 months (95% CI: 35.196-65.42), with a difference of approximately 16 months (p = 0.008) (Table 5 and Figure 5).

Survival analysis according to cutoff values for overall survival

**Figure 6:**
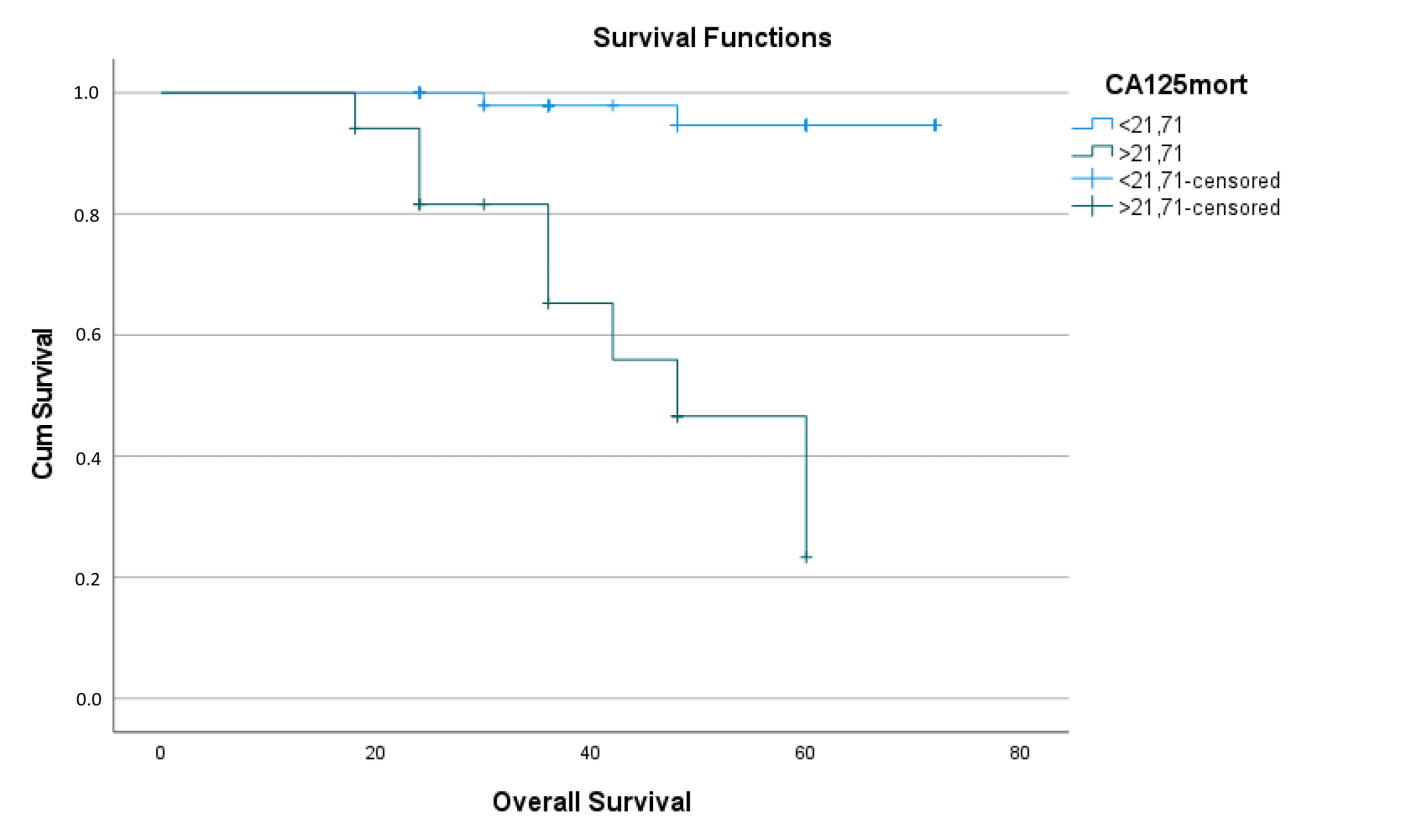
Kaplan-Meier survival analysis function graph of CA 125 for overall survival CA125mort: Cancer antigen 125 cut-off groups for mortality

The Kaplan-Meier survival analysis and the log-rank test indicated that the mean estimated overall survival expectancy for the whole cohort was 65.58 ± 1.87 months (95% CI: 61.922-69.250). Patients with CA 125 level < 21.71 had a mean estimated overall survival time of 70.37 ± 1.15 months (95% CI: 68.094-72.59), while those with CA 125 level ≥ 21.71 had a mean estimated overall survival time of 46.30 ± 4.18 months (95% CI 38.105-54.49), with a difference of approximately 26 months (p < 0.001) (Table 6). At 60 months, the estimated overall survival rate was approximately 95% in the CA 125-negative group, decreasing to nearly 25% in the CA 125-positive group (Figure 6).

**Table 6:** Estimated durations for the overall and disease-free survival stratified for CA 125, CAR, and CEA cutoff values CI: confidence interval, CA 125: cancer antigen 125, CAR: C-reactive protein/albumin ratio, *: Estimated with the Haldane-Anscombe correction technique

Variables	Cutoff	Estimated Overall Survival Duration (months)	Std. Error	95% CI	Log Rank test Statistical Significance
CA125 for Overall Survival Duration	<21.71	70.34	1.15	68.094-72.59	<0.001
	≥21.71	46.30	4.18	38.105-54.49	
CAR for Overall Survival Duration	<4.09	70.62*	1.36*	67.954-73.27*	0.007*
	≥4.09	60.11*	3.03*	54.203-66.02*	
Estimated Overall Survival Duration (All Participants)	x	65.58	1.87	61.922-69.250	x

**Figure 7:**
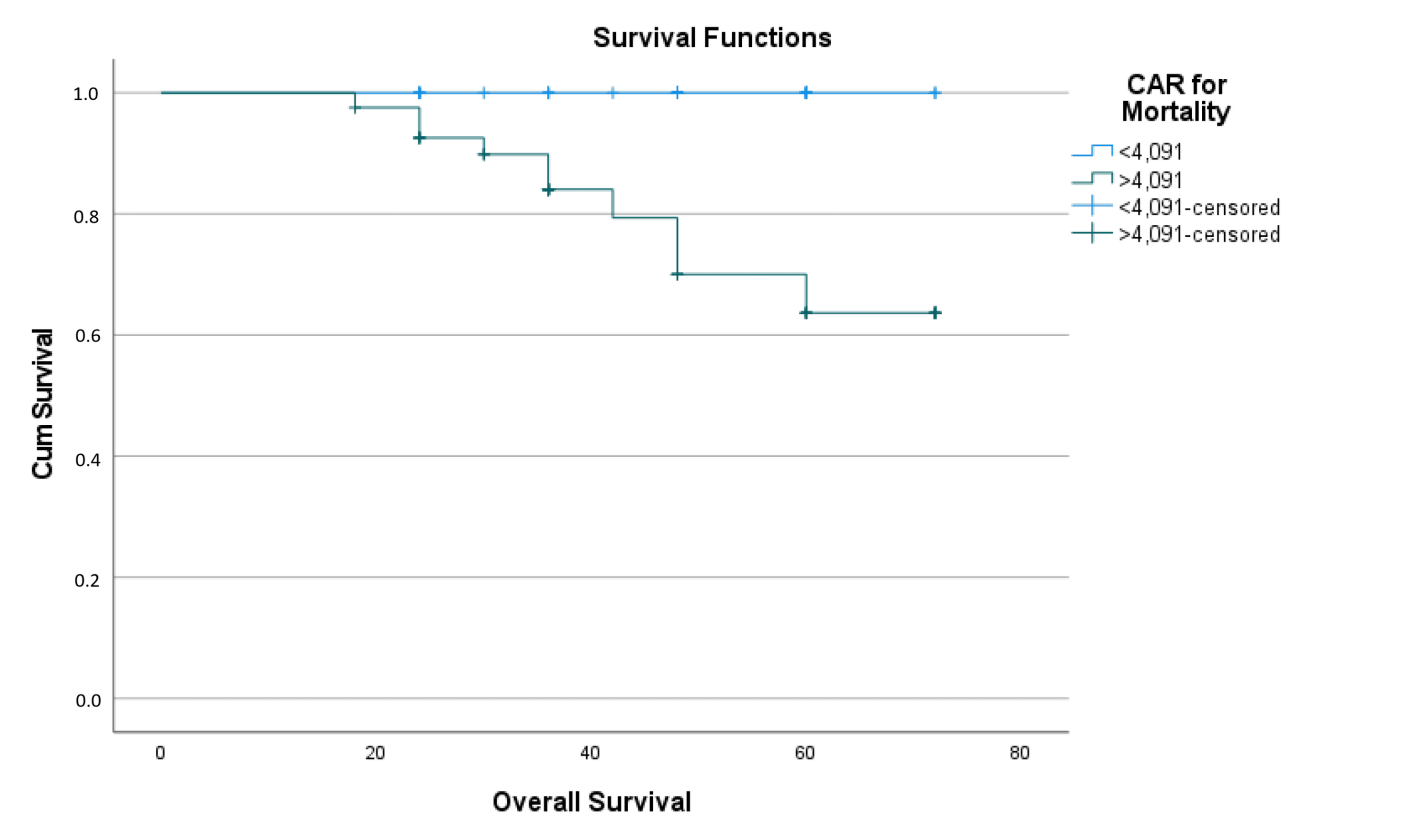
Kaplan-Meier survival analysis function graph of CAR for overall survival CAR: C-reactive protein/albumin ratio

Patients with CAR level < 4.09 had a mean estimated overall survival time of 70.62 ± 1.36 months (95% CI: 54.203-66.02), while those with CAR level ≥ 4.09 had a mean estimated overall survival time of 60.11 ± 3.03 months (95% CI: 54.203-66.06), with a difference of approximately 10 months (p = 0.001; the Haldane-Anscombe correction technique was used for estimations) (Table 6 and Figure 7).

## Discussion

Our study provides significant insights into the prognostic factors influencing survival and recurrence in patients over a follow-up period ranging from 18 to 72 months. The findings highlight the critical role of patient age, disease recurrence, and specific biomarkers in determining patient outcomes.

Colonic adenocarcinoma remains a major health issue, being one of the most prevalent malignant tumors of the colon [15]. It affects more than 70,000 men and 60,000 women annually, with over 600,000 deaths attributed to this disease [15]. As observed in the literature, the majority of our patients were male (63.4%), and a significant proportion (60.56%) had additional systemic diseases, including diabetes mellitus, hypertension, or asthma. Despite this similarity, comorbidities and gender distribution were not significantly different between the surviving and deceased groups, indicating that gender or other comorbidities alone did not influence mortality in our cohort. A study by Rodrigues et al. [16] found that mortality rates for colon cancer are higher in men than in women, but this pattern was not observed in our patient group.

Age is a well-established risk factor for mortality in colon cancer [17,18]. Our analysis also revealed that age is a significant determinant of mortality in colon cancer patients. The mean age of deceased patients was notably higher than that of surviving patients (74.5 ± 12.27 years vs. younger patients, p = 0.026), suggesting that advancing age may exacerbate underlying vulnerabilities, leading to poorer outcomes. This finding is consistent with existing literature, which consistently identifies age as a crucial factor in patient prognosis, particularly in chronic and severe illnesses [17-19].

The recurrence of colon cancer has a significant impact on mortality, with studies highlighting the association between cancer recurrence and increased risk of death [20]. Zhou et al. [21] emphasized that a large number of patients, even after curative surgery, still face a high risk of disease recurrence, which is a major cause of cancer mortality. A study by Huynh Thanh et al. [22] highlighted that the recurrence rate after surgery for colon cancer is approximately 29% in patients with stage 2 cancer and 42% in those with stage 3 cancer. While we observed similar results in stage 2 cancer recurrence, the recurrence rate in our cohort was higher for stage 3 colon cancer patients. In our study disease recurrence emerged as a major predictor of both mortality and reduced disease-free survival. The recurrence rate was significantly higher in the deceased group (60% vs. 8.2%, p < 0.001), and overall survival was significantly shorter in the recurrent disease group, indicating a strong correlation between recurrence and poor outcomes as expected [23].

Inflammatory markers, such as CRP and CAR, are known to be elevated in patients with adverse clinical outcomes in colon adenocarcinoma [5,24-27]. Elevated CRP levels are indicative of systemic inflammation, which is known to contribute to cancer progression by enhancing tumor growth, invasion, and metastasis [24]. Research has consistently demonstrated a correlation between high CRP and CAR levels and advanced disease stages, increased recurrence rates, and reduced survival in patients with colon adenocarcinoma [5,26]. CAR is increasingly regarded as a significant prognostic marker in many clinical situations [28]. An elevated CAR reflects both heightened inflammation (as indicated by high CRP) and compromised nutritional status (as reflected by low albumin), both of which are associated with adverse outcomes in cancer patients [28]. Studies have linked higher CAR values with poorer overall survival and disease-free survival in colon adenocarcinoma, underscoring its utility in risk stratification and informing treatment strategies [25-27]. In our study, inflammatory markers, particularly CRP and CAR, were found to be significantly elevated in patients with poorer outcomes. CRP levels were notably higher in the deceased group (p=0.003), and CAR levels were significantly elevated in both deceased and recurrent disease groups (p = 0.001 and p = 0.045, respectively). These findings support the growing body of evidence that chronic inflammation plays a pivotal role in disease progression and could serve as a valuable prognostic tool in clinical practice [26].

CEA is a glycoprotein tumor marker that has been extensively studied in various medical conditions, particularly in the context of cancer. CEA was first described as a specific antigen present in both fetal colon and colon adenocarcinoma in 1965 [6,29]. It is widely used for diagnosing various cancers, especially colon adenocarcinoma [30]. CEA is considered a tumor-associated antigen of endodermally derived tissue due to its expression on a variety of neoplasms, particularly those of the gastrointestinal tract [31]. The antigen is known to be expressed in adenocarcinomas of the colon, stomach, pancreas, breast, lung, and other organs [14]. Additionally, CEA has been shown to act as a homotypic adhesion molecule, forming CEA-CEA bridges, and modulating intercellular adhesion, suggesting its role as a facilitator of tumor invasion and metastasis [13]. Studies have demonstrated the prognostic effect of preoperative CEA levels and the usefulness of postoperative CEA monitoring for early detection of recurrence after curative surgery and for assessing the response to chemotherapy in metastatic colorectal cancer [32]. Among the tumor markers we evaluated in our study, CEA and CA 125 were particularly noteworthy. Elevated levels of CEA were strongly associated with disease recurrence (p = 0.001), while higher CA 125 levels were linked to both recurrence and overall mortality. The significant associations between these tumor markers and patient outcomes suggest that they could be instrumental in risk stratification and in guiding post-operative surveillance strategies [4,33,34].

CA 125, also known as cancer antigen 125, is a glycoprotein tumor marker [35]. This glycoprotein is highly glycosylated and is secreted by mesothelial cells, which can lead to elevated levels in the blood due to peritonitis or inflammation that disrupts blood vessels, causing CA 125 to accumulate [35]. Studies have shown that CA 125 plays a significant role in diagnosing and monitoring various cancers, such as ovarian cancer, where it has been used as a marker for detecting and managing epithelial ovarian cancer for several decades [33]. Elevated serum CA 125 levels have been associated with various conditions, including liver cirrhosis, endometriosis, and malignant lymphomas, indicating its versatility as a biomarker in different medical contexts [36-38]. The ROC analysis further reinforced the prognostic value of CEA, CA 125, and CAR. For instance, a CEA cutoff value of ≥ 47.145 was highly specific (98.3%) for predicting recurrence, while a CA 125 cutoff value of ≥ 21.715 demonstrated high sensitivity (80%) and specificity (85.2%) for predicting mortality. A similar study by Huang et al. [8] has found that patients with elevated preoperative CA 125 levels above 35 U/mL were found to have a poorer prognosis after surgery than those with normal preoperative CA 125 levels. This was 21.715 U/mL for our cohort. We believe that these cutoff points can offer practical thresholds for clinicians to assess patient risk more accurately.

Kaplan-Meier survival analysis confirmed that elevated levels of CEA, CA 125, and CAR are associated with significantly reduced disease-free and overall survival times. Patients with CEA levels below the threshold of 47.145 had a mean estimated disease-free survival time of 67.73 months, compared to only 24 months in those with higher levels (p < 0.001). Similarly, lower CA 125 and CAR levels correlated with longer overall survival times, reinforcing the utility of these markers in predicting long-term prognosis. Patients with CA 125 < 21.71 had a mean estimated overall survival time of 70.37 ± 1.15 months, while those with CA 125 ≥ 21.71 had a mean estimated overall survival time of 46.30 ± 4.18 months, with a difference of approximately 26 months. This difference was also existent but less than our calculations with an 18-month difference in the study done by Huang et al. [8].

The findings from this study suggest that older age, disease recurrence, and elevated levels of CEA, CA 125, and CAR are strong predictors of adverse outcomes. These factors should be closely monitored in clinical practice to identify high-risk patients who may benefit from intensified surveillance and therapeutic strategies. Additionally, the identified cutoff values for these biomarkers offer practical thresholds that can guide clinical decision-making, ensuring that high-risk patients receive appropriate interventions.

This study, while providing valuable insights, is subject to several limitations that warrant consideration. First, the retrospective design inherently limits the ability to establish causal relationships. Second, the study was conducted at a single institution, which may limit the generalizability of the findings to broader populations. The relatively small sample size of 71 patients further restricts the statistical power of the analysis, particularly when subgroup comparisons are made. Another limitation is the exclusion of patients with any medication use, hematologic diseases, or active infections that could alter hemogram and biochemistry parameters. While this criterion was necessary to maintain the homogeneity of the sample, it may also limit the applicability of the results to patients with more complex clinical presentations, which are common in real-world settings. Finally, while sophisticated statistical methods, including ROC curve analysis and Kaplan-Meier survival analyses, were employed to evaluate the prognostic significance of various biomarkers, the study's findings should be interpreted with caution, given the retrospective nature of the data. Prospective, multicenter studies with larger sample sizes are needed to validate these findings and further explore the clinical utility of the identified biomarkers in predicting outcomes for patients with colonic adenocarcinoma.

## Conclusions

In conclusion, these data highlight the potential of CEA and CA 125, and we suggest that CEA may be a useful tool for predicting the recurrence of colon adenocarcinoma and estimating disease-free survival durations in patients who have undergone surgery. Furthermore, CA 125 may be a valuable indicator for predicting mortality and estimating overall survival duration in this patient population. These factors collectively provide a robust framework for predicting patient outcomes and tailoring treatment strategies to improve survival and quality of life. Future research should continue to explore the mechanistic links between these biomarkers and disease progression, as well as validate the clinical utility of the identified cutoff values in larger, more diverse populations.
